# High-dose vitamin C intravenous infusion in the treatment of patients with COVID-19: A protocol for systematic review and meta-analysis

**DOI:** 10.1097/MD.0000000000025876

**Published:** 2021-05-14

**Authors:** Lifang Huang, Lang Wang, Jianghong Tan, Hong Liu, Yanhui Ni

**Affiliations:** a Department of metabolic Endocrinology; b Nursing Department; c Integrated TCM & Western Medicine Department, Zhuzhou Central Hospital, Zhuzhou, Hunan province, China.

**Keywords:** COVID-19, L-ascorbic acid, meta-analysis, protocol, systematic review, vitamin C

## Abstract

**Background:**

Patients infected with a virus usually lack vitamin C. High-dose vitamin C has an antiviral effect, and has been used by several researchers to treat COVID-19 by intravenous infusion, achieving good results. However, the efficacy and safety of vitamin C in the treatment of patients with COVID-19 remain unclear. Thus, the aim of the present study was to investigate the efficacy of high-dose vitamin C infusion in the treatment of patients with COVID-19.

**Methods:**

Electronic databases were searched, including PubMed, EMBASE, Cochrane Central Register of Controlled Trials, Web of Science, China National Knowledge Infrastructure database, Chinese Wanfang database, and Chinese Biomedical Literature database. The aim was to collect randomized controlled trials of high-dose vitamin C infusion in the treatment of patients with COVID-19, with the retrieval time being from the establishment of the database to March 2021. In accordance with the pre-designed inclusion/exclusion criteria, all data were extracted independently by 2 researchers. To assess the risk bias in the studies, the Cochrane collaboration's tool for assessing risk of bias was used to assess the risk bias in the studies, while meta-analysis was performed using Revman 5.3 software.

**Results:**

In the present study, a high-quality comprehensive evaluation is provided of high-dose vitamin C infusion in the treatment of patients with COVID-19.

**Conclusion:**

Further convincing evidence for the clinical treatment of COVID-19 is provided, in addition to evidence-based guidance for clinical practice.

**PROSPERO Registration Number:**

CRD42021246342.

## 1 Introduction

The outbreak of the novel coronavirus (COVID-19) began in December 2019, and remains a prevalent threat around the world. COVID-19 is highly infectious and has high mortality, especially in patients with underlying health issues (such as diabetes).^[1]^ At present, there is no specific drug for the treatment of COVID-19, and many potential therapeutic drugs have been included in the scope of clinical trials. Several institutions have focused on verifying the therapeutic effect of vitamin C on COVID-19.^[2,3]^

Also known as L-ascorbic acid, vitamin C is a kind of water-soluble vitamin that exists in blood and cells in the form of reduced ascorbic acid. Clinically, vitamin C is mainly used for the treatment of scurvy, and also for the adjuvant treatment of various acute and chronic infectious diseases.^[4]^ Vitamin C is an essential vitamin for human immune system, which can enhance the body's immunity to viruses in a number of ways. High concentration of vitamin C can enhance the antiviral ability, especially in the form of dehydroascorbic acid, which can inhibit influenza A virus.^[4,5]^ As revealed by prior research, vitamin C has a strong antiviral effect only in large doses,^[6]^ and serum vitamin C levels are low in most critically ill patients with COVID-19.^[7,8]^ Hence, the use of high-dose vitamin C in the treatment of patients with COVID-19 is certainly feasible.

Despite the feasibility, there is a scarcity of high-quality evidence to support the efficacy and safety of high-dose vitamin C in patients with COVID-19. For this reason, in the present study, the efficacy of vitamin C therapy was systematically evaluated, so as to provide evidence-based guidance for clinical application.

## 2 Materials and methods

### 2.1 Study registration

The present study was conducted in accordance with the Preferred Reporting Items for Systematic Reviews and Meta-analysis Protocols statement guidelines,^[9]^ and has been registered with PROSPERO under the registration number: CRD42021246342.

### 2.2 Selection criteria

#### 2.2.1 Type of studies

The present study is a randomized controlled trial of high-dose vitamin C infusion in the treatment of COVID-19.

#### 2.2.2 Types of patients

Inclusion criteria:

1. Patients diagnosed with COVID-19;
2. Patients with a mini-mental state examination score > 21;
3. Patients of 18 years old or above.

Exclusion criteria:

1. Patients with heart failure;
2. Patients with renal failure;
3. Patients with liver failure;
4. Pregnant and lactating women.

#### 2.2.3 Types of interventions and comparisons

Treatment group: high dose vitamin C intravenous infusion, daily total amount ≥10 g. Control group: the placebo group received bacteriostatic water for injection in the same way, or a small dose of vitamin C, daily total amount ≤2 g.

#### 2.2.4 Types of outcomes

1. PaO_2_/FiO_2_;
2. Invasive mechanical ventilation-free days in 28 days;
3. interleukin-6;
4. 28-day mortality;
5. Length of stay;
6. Adverse event rate.

### 2.3 Exclusion criteria

1. Articles without full-text access or articles that cannot extract data;
2. Studies published repeatedly;
3. Reviews, systematic reviews, abstracts, conferences, dissertations, animal experiments, and other literature.

### 2.4 Search strategy

The electronic databases searched include PubMed, EMBASE, Cochrane Central Register of Controlled Trials, Web of Science, China National Knowledge Infrastructure database, Chinese Wanfang database and Chinese Biomedical Literature database. The retrieval time was from the establishment of the database to March 2021. Taking PubMed as an example, the retrieval strategy is shown in Table 1.

**Table 1:** Search strategy in PubMed database.

Number	Search terms
#1	Corona Virus Disease 2019 [Title/Abstract]
#2	Novel coronavirus [Title/Abstract]
#3	COVID-19 [Mesh]
#4	SARS-CoV-2[Mesh]
#5	#1 OR #2 OR #3 OR #4
#6	VC[Title/Abstract]
#7	Vitamin C [Title/Abstract]
#8	L-Ascorbic Acid [Title/Abstract]
#9	L Ascorbic Acid[Title/Abstract]
#10	Ascorbic Acid [Mesh]
#11	#6 OR #7 OR #8 OR #9 OR #10
#12	Intravenous infusion [Title/Abstract]
#13	Intravenous drip [Title/Abstract]
#14	Drip, Intravenous [Title/Abstract]
#15	Infusions, Intravenous [Mesh]
#16	#12 OR #13 OR #14 OR #15
#17	#5 AND #11 AND #1

### 2.5 Study selection and data extraction

#### 2.5.1 Selection of studies

**Figure 1:**
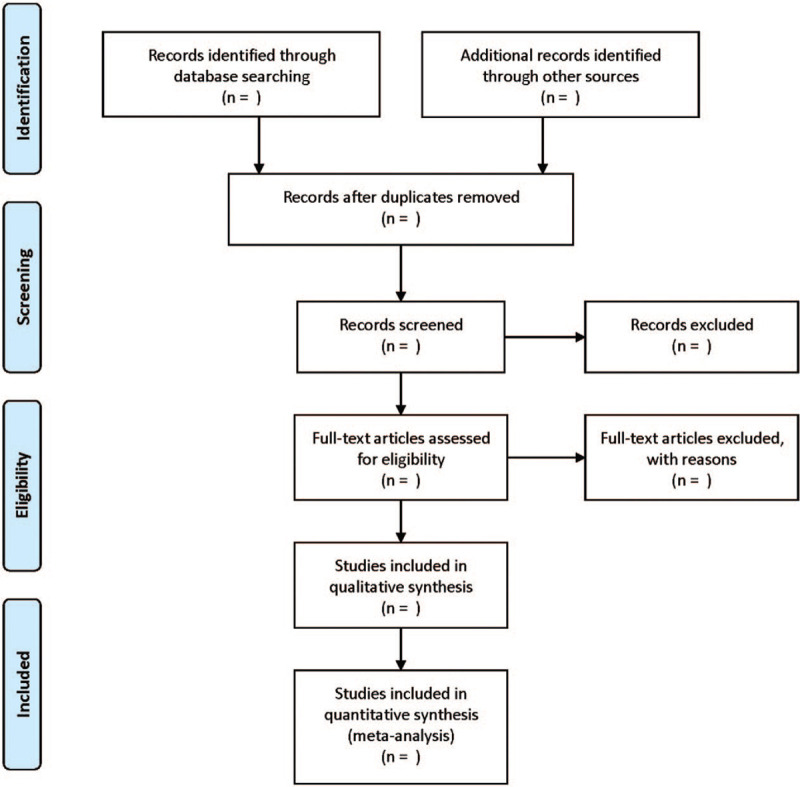
Flow diagram of study selection process.

Two researchers searched independently, with the literature being independently screened and evaluated according to the inclusion and exclusion criteria. If an article could not be determined, the 2 researchers would make a decision or discuss with a third researcher. The detailed literature selection process is shown in Figure 1.

#### 2.5.2 Data extraction

Two researchers independently extracted and examined the data, which included the year of publication, region of publication, patient age, patient gender, sample size, drug dosage, control measures, study period, outcome indicators, and others. Any problems of missing data were solved by contacting the corresponding author or through intention analysis.

#### 2.5.3 Assessment of risk of bias

The Cochrane collaboration's tool was used to assess the risk bias in the included studies, which were independently evaluated by the 2 researchers. If there were differences in the evaluation results, the 2 researchers would discuss with each other or negotiate with the third researcher.

#### 2.5.4 Measures of treatment effect

For continuous variables, the mean difference and the 95% confidence intervals thereof were used; For categorical variables, the risk ratio and the 95% confidence intervals thereof were used.

#### 2.5.5 Data analysis and heterogeneity processing

The data analysis of the present study was conducted through Review Manager Version 5.3 software (The Cochrane Collaboration, Oxford, England). The heterogeneity among the studies was judged according to Q-test and *I*^2^. When *P* ≥ .1, *I*^2^ ≤ 50%, this indicated that there was homogeneity among the studies. The fixed effect model was used for analysis,^[10]^ and when *P* ≥ .1, *I*^2^ > 50%, this indicated that there was heterogeneity among the studies. In this case, the source of heterogeneity should be found. Subgroup analysis or sensitivity analysis was performed on the factors that may lead to heterogeneity. A random effects model was used in cases where there was statistical heterogeneity among the studies, but no clinical or methodological heterogeneity.^[11]^ If the heterogeneity was too large and the source of heterogeneity could not be determined, only descriptive analysis was conducted.

#### 2.5.6 Subgroup analysis

Subgroup analysis was performed according to the dose of vitamin C (<20 g or ≥ 20 g).

#### 2.5.7 Sensitivity analysis

The stability of the meta-analysis results was tested through a one-by-one elimination method in sensitivity analysis.

#### 2.5.8 Assessment of reporting bias

A funnel plot was used to detect publication bias, while the Egger test of bias was used as a supplement.^[12]^

#### 2.5.9 Ethics and dissemination

The rights of participants were not endangered in the present systematic evaluation, and ethical approval was not required. Research results may be published in a peer-reviewed journal or disseminated in relevant conferences.

## 3 Discussion

Vitamin C has been extensively applied in the treatment of viral infections, with studies revealing that patients with pneumonia and sepsis have low levels of vitamin C and elevated oxidative stress.^[13]^ Owing to the direct inhibitory effect thereof on pathogens, there is sufficient evidence to suggest that vitamin C is effective in the treatment of pneumonia and infection.^[14]^ Further, vitamin C has been shown to reduce the severity and duration of pneumonia,^[15]^ while meta-analysis of 12 trials in 1766 patients calculated that vitamin C reduced ICU stay by an average of 8%.^[16]^ Another meta-analysis found that vitamin C reduced the duration of mechanical ventilation in ICU patients.^[17]^ Novel coronavirus pneumonia is a new respiratory disease caused by COVID-19, and is severely infectious.^[18]^ Thus, vitamin C novel coronavirus pneumonia can also be applied to the treatment of patients.

Vitamin C can improve the resistance of white blood cells to viruses, has an antioxidant effect, and can induce interferon production in vivo.^[4,19]^ In viral infections, vitamin C can attenuate pro-inflammatory response, enhance epithelial barrier function, increase alveolar fluid clearance rate, and prevent sepsis related coagulation abnormalities.^[20]^ The implementation of high-dose vitamin C treatment can significantly reduce the demand for high-dose corticosteroids, antibiotics, and antiviral drugs, as these drugs may have the effects of immunosuppression, adrenal suppression and toxicity, complicating the course of the disease.^[21]^ Combined with traditional Chinese medicine, Yali used a large dose of vitamin C (20 g/60 kg per day) to treat COVID-19.^[22]^ As a result of Yali treatment, the symptoms of fatigue, cough, dry throat, and shortness of breath were significantly improved, and no adverse events occurred.^[22]^ Zhang used a large dose of vitamin C (24 g/day) at the rate of 12 mL/h to treat patients with COVID-19,^[23]^ with the results revealing that the PaO_2_/FiO_2_ of patients increased steadily, and the 28 day mortality of patients decreased significantly.^[23]^ Therefore, high-dose vitamin C infusion may be a significantly effective therapeutic agent in COVID-19 treatment.

Since there is currently a scarcity of related research, whether high-dose vitamin C can improve the prognosis of patients with COVID-19 is yet to be ascertained, and is a high-priority concern for medical scholars. The present study is the first systematic review and meta-analysis of the efficacy of high-dose vitamin C in patients with COVID-19. Notably, the lack of adequate randomized controlled trials could be regarded as a limitation of the present meta-analysis.

## Author contributions

**Conceptualization:** Lifang Huang.

**Data collection:** Lang Wang, Jianghong Tan.

**Funding acquisition:** Jianghong Tan.

**Resources:** Hong Liu.

**Software:** Lang Wang.

**Supervision:** Yanhui Ni.

**Writing – original draft:** Lifang Huang, Lang Wang and Yanhui Ni.

**Writing – review & editing**: Lifang Huang, Jianghong Tan and Yanhui Ni.
